# Difficulties in the Management of Placenta Accreta Spectrum Disorders are not Confined to Low-/Middle-Income Countries: A Possible Usefulness of Simulation Training

**DOI:** 10.1055/s-0042-1751073

**Published:** 2022-09-08

**Authors:** Kenro Chikazawa, Shigeki Matsubara, Tomoyuki Kuwata

**Affiliations:** 1 Department of Obstetrics and Gynecology, Saitama Medical Center, Jichi Medical University, Japan; 2 Department of Obstetrics and Gynecology, Koga Red Cross Hospital, Japan

Dear Editor,

Aguilera et al. 1 clearly illustrated some difficulties in the management of placenta accreta spectrum (PAS) disorders in low-/middle-income countries (LMICs), with special emphasis on the low rate of presurgical diagnosis. This naturally forced obstetric surgeons to handle “unexpected PAS disorders” on an emergency basis, and this situation may also be true in high-income countries (HICs).

The recommendation of Aguilera et al. 1 is to provide periodic education for professionals who may have to deal with PAS disorders, and we believe that this is true in both LMICs and HICs. Therefore, we believe that simulation training might become an important tool.

Simulation should cover what to do when encountering “unexpected PAS disorders” during delivery/surgery for the following reasons: first, the surgery for PAS disorders is still life-threatening. 2 A study 2 has shown that 82 deaths were associated with surgery for PAS disorders during a 5-year period in 16 countries on 3 continents. Importantly, the insufficient experience/knowledge on the part of the surgeons accounted for many of the deaths. 2

Secondly, even though in many HICs planned surgery for diagnosed cases may now be performed by a multidisciplinary team in a well-prepared manner, presurgical diagnosis of PAS disorders is still difficult, so not all PAS disorders are diagnosed preoperatively. 2 Aguilera et al. 1 reported that approximately a half of the surgeries were performed at night; this may be an extreme, but any obstetrician, including those less experienced, might encounter it incidentally during cesarean section.

Third, PAS disorders are not common; therefore, not every obstetrician will observe cases of them in their practices. The simulation training hitherto focuses on “common procedures” such as laparoscopic surgery, vacuum or forceps delivery, or cosmetics-oriented skin closure, which are all “everyday-practice procedures”. That may be the reason why simulation training for PAS disorders has been less frequently provided.

Surgery for PAS disorders requires experienced hands. 3 4 One may not be able to perform it solely based on simulation training. However, since any obstetrician might encounter a patient with an undiagnosed PAS disorder during cesarean section, for example, and since inexperience cannot become an excuse for an inability to handle the situation, obstetricians may benefit from simulation training on how to deal with an unexpected PAS disorder on an emergency basis.

It is beyond the scope of this letter to discuss what is required for the training and how to provide it. However, we believe that the following may become goals of the simulation training; trainees can: 1) intra-surgically diagnose the condition as placenta percreta: understand that the following two strongly indicate very severe conditions, i.e., the placenta being discernable from the uterine serosa and the presence of engorged vessels; 2) know how and when to add uterine compression sutures; 3) effectively insert an intrauterine hemostatic balloon; 4) perform compression of the abdominal aorta compression; and 5) know how and when to perform hysterectomy. Regarding hysterectomy, subtotal hysterectomy or an amputation-first procedure 3 may be sufficient, considering the emergency and depending on the situation. Simulation is expected to be performed both at an individual or team level and also scientific-meeting level, because with the management of an unexpected PAS disorder should be understood and shared by individuals, teams, and obstetric societies. Some internet-based educational tools (including ours, regarding Bakri-balloon-insertion) 5 may be useful additions to the simulation training. Bearing the final goals of simulation training in mind, one can create/modulate/devise the actual simulation program depending on the situations that are specific to each region of the world.

The good patient outcomes shown by Aguilera et al. 1 have impressed us: lower levels of massive bleeding, lower rate of surgical complications, and, importantly, no maternal deaths. The authors 1 considered that this was due to “experienced hands”: the obstetric surgeons in their hospital had no choice but to perform many difficult surgeries; thus, they were used to performing cesarean hysterectomy for PAS disorders. However, we believe that no surgeon begins their career with “experienced hands”. Patients may die during surgery for PAS disorders. One may not have experienced hands, but, depending on the situation, be obliged to perform surgery for PAS disorders regardless of this. Simulation training may be of great help, and much discussion is needed as to how, when, and to whom the training should be provided.
